# Ideal vs Actual Timing of Palliative Care Integration for Children With Cancer in Latin America

**DOI:** 10.1001/jamanetworkopen.2022.51496

**Published:** 2023-01-19

**Authors:** Michael J. McNeil, Bella Ehrlich, Huiqi Wang, Marisol Bustamante, Veronica Dussel, Paola Friedrich, Ximena Garcia Quintero, Srinithya R. Gillipelli, Wendy Gómez García, Dylan Graetz, Erica C. Kaye, Monika Metzger, Carla Vittoria Sabato Danon, Meenakshi Devidas, Justin N. Baker, Asya Agulnik

**Affiliations:** 1 Department of Global Pediatric Medicine, St Jude Children’s Research Hospital, Memphis, Tennessee; 2 Division of Quality of Life and Palliative Care, Department of Oncology, St Jude Children’s Research Hospital, Memphis, Tennessee; 3 Brown University School of Medicine, Providence, Rhode Island; 4 Universidad de San Carlos de Guatemala, Guatemala City, Guatemala; 5 Center for Research and Implementation in Palliative Care, Buenos Aires, Argentina; 6 Fundación Valle de Lilli, Cali Colombia; 7 School of Medicine, Baylor College of Medicine, Houston, Texas; 8 Dr Robert Reid Cabral Children’s Hospital, Santo Domingo, Dominican Republic; 9 Centro de Diagnóstico y Terapia Psicológica, Mexico City, Mexico

## Abstract

**Importance:**

Early integration of pediatric palliative care (PPC) for children with cancer is critical for the quality of life of both patient and family. To improve access to PPC in resource-limited settings, barriers to early integration must be understood.

**Objectives:**

To evaluate the ideal vs actual timing of PPC integration for children with cancer and to uncover barriers to early integration identified by physicians in Latin America.

**Design, Setting, and Participants:**

The Assessing Doctors’ Attitudes on Palliative Treatment (ADAPT) survey was distributed electronically from August 1, 2020, to January 31, 2021, to physicians who treat children with cancer in 17 countries in Latin America.

**Main Outcomes and Measures:**

The ADAPT survey queried for understanding of ideal vs actual timing of PPC for children with cancer and for identification of barriers to PPC integration. Descriptive statistics were used to summarize the data. For secondary analyses, a comparison of the associations of previous palliative care training with physician specialty was performed using the Pearson χ^2^ test or the Fisher exact test. The McNemar test was used to assess responses regarding the actual vs ideal timing of PPC consultation. Analysis of variance was used to compare mean values for perceived barriers by country income level. Answers to open-ended questions were analyzed qualitatively.

**Results:**

A total of 831 physicians (578 women [69.6%]; 275 physicians [33.1%] aged <35 years and 556 physicians [66.9%] aged ≥35 years) from 17 countries participated, with an overall response rate of 37.9% (831 of 2193) and a median country response rate of 51.4% (range, 22.2%-88.9%). Most respondents (572 [68.8%]) said that PPC should be involved from diagnosis, but only 117 (14.1%) stated that this occurred at their institution (*P* < *.*001)*.* The most significantly ranked barriers to PPC were lack of home-based services (713 [85.8%]), personnel (654 [78.7%]), and knowledge about PPC (693 [83.4%]), along with physician (676 [81.3%]) and family (603 [72.6%]) discomfort about PPC involvement. In addition, these barriers were rated as more important in lower-middle income countries compared with upper-middle income countries and high-income countries.

**Conclusions and Relevance:**

This study highlights the discrepancy between ideal and actual timing of PPC for children with cancer and barriers to early PPC integration in Latin America. Interventions addressing access to PPC resources, didactic training, and clinical education (with a particular focus on equitable access to basic resources and support) are critical to improve the timing and quality of PPC in the region.

## Key Points

**Question:**

What is the ideal timing for palliative care consultation for children with cancer in Latin America, and what are the barriers to earlier integration of palliative care in patient treatment?

**Findings:**

A total of 831 physicians from 17 countries participated in this survey-based study; most respondents stated that the ideal timing for palliative care was much earlier than what is currently happening. Significant barriers included lack of home-based care, limited physician knowledge, discomfort with palliative care, and family resistance.

**Meaning:**

Physicians in Latin America identified earlier palliative care integration as important for children with cancer, but this study found a difference between ideal timing and actual timing of palliative care integration, highlighted by specific barriers to this earlier integration.

## Teaser

This survey study evaluates the ideal vs actual timing of palliative care integration for children with cancer and assesses barriers to early integration identified by physicians in Latin America.

## Introduction

The World Health Organization (WHO) emphasizes early integration of palliative care as a “medical and moral necessity” for children with life-threatening illnesses and their families.^1^ Access to palliative care should be a part of treatment regardless of resources or clinical prognosis.^1,2^ The early integration of pediatric palliative care (PPC) improves pain and symptom management and quality of life for patients, reduces the suffering of patients and their caregivers, and improves communication between health care professionals and families.^1^

Between 80% and 90% of the burden of global childhood cancer remains in low- and middle-income countries (LMICs), where fewer than 1 in 4 survive their disease, as opposed to survival rates higher than 80% in some high-income countries (HICs).^3,4,5,6^ Early integration of PPC has been shown to provide significant benefits for children with cancer. This earlier integration results in improved communication between health care professionals and families, reduces patient and caregiver suffering, and improves pain and symptom management for patients.^1,7,8,9^ Therefore, the WHO Global Initiative for Childhood Cancer advocates for palliative care integration as a “core component of comprehensive care starting when illness is diagnosed.”^10^ Despite this need for PPC for children with cancer in resource-constrained settings, access is inadequate. More than 65% of countries worldwide lack the capacity to provide PPC,^11^ and countries with the highest PPC needs are those with the least capacity for PPC provision.^12,13,14^

Several barriers impede the development and integration of PPC for children with cancer, including prognostic uncertainty, unrealistic expectations of cure, lack of physician understanding of palliative care, and perceived parental refusal.^15,16,17,18,19^ Most studies identifying these barriers were conducted in HICs, but there is growing literature on additional structural and health care system issues faced in LMICs, including limited access to trained personnel, financial constraints, and sociocultural factors.^12,13,14^

When considering provision of PPC for children with cancer, it is important to differentiate between specialized and primary palliative care.^20^ Specialty palliative care is the provision of palliative care to patients by professionals who have received additional training and work in conjunction with a patient’s primary team. Primary palliative care can be provided by any health care professional and not those who received additional subspecialty training. Access to specialty palliative care professionals can be difficult in LMICs and is another barrier to the early integration of PPC for children with cancer.

Latin America is a large and heterogenous continent with a range of cultures and resources in care for children with cancer. Depending on the country, health system, and hospital structure, the diagnosis, management, and care of a child with cancer may vary greatly. Even within a country, care and access vary greatly depending on whether the institution is in a rural or urban center or is a private or public institution.^21^ However, concerted efforts are being made to address barriers to cancer care for children, including high-quality PPC, such as interventions in education and capacity building, along with advocacy efforts through national and international organizations.^22,23,24,25^ Improving PPC in Latin America requires research to identify specific barriers to early integration of PPC for children with cancer in the region.

In collaboration with St Jude Global and the WHO Global Initiative for Childhood Cancer,^26,27^ the Assessing Doctors’ Attitudes on Palliative Treatment (ADAPT) survey was originally created to evaluate physician perspectives on PPC integration into childhood cancer care in Eurasia.^28,29^ This survey was adapted and translated into Spanish and distributed among physicians in 17 countries in Central and South America, with analysis demonstrating that physicians had a general understanding of PPC but were uncomfortable addressing the PPC needs of their pediatric patients with cancer.^25^ In this secondary analysis, we assess the ideal vs actual timing of palliative care and the barriers to early integration of PPC for children with cancer in Central and South America.

## Methods

### Instrument Development or WHO Alignment

The ADAPT survey was originally developed to better understand the perceptions of physicians who care for children with cancer on early integration of PCC.^1,19,28,29,30,31,32^ The methods for the adaptation of the ADAPT survey for Latin America has been previously described.^25^ In brief, the survey was translated into Spanish and evaluated by an expert panel to ensure items were consistent with the cultural context. The survey was subsequently back-translated to English to confirm construct consistency.^33^ After piloting, the final survey contained 65 questions, with 62 closed-ended items using a 5-point Likert scale (where 1 indicates “strongly disagree” and 5 indicates “strongly agree”) or multiple-choice format with 3 open-ended questions (eAppendix in Supplement 1). The Office of Human Subjects Research Protections and institutional review board at St Jude Children’s Research Hospital, Memphis, Tennessee, approved this study as exempt research because the participants’ identity could not be readily ascertained. Additional approvals by participating institutions were obtained as needed. Voluntary completion of the survey was considered consent to participate in the study. The development of the survey was performed following the best practice established by the American Association for Public Opinion Research (AAPOR) reporting guideline.^34^ This publication focuses on questions assessing ideal vs actual timing of PPC and the perceived barriers to earlier integration of PPC for children with cancer.

### Instrument Distribution Strategy

The survey was distributed anonymously via the Qualtrics electronic software platform^35^ to physicians who treat children with cancer in 17 participating countries: Argentina, Bolivia, Chile, Colombia, Costa Rica, Dominican Republic, Ecuador, El Salvador, Guatemala, Haiti, Honduras, Mexico, Nicaragua, Panama, Paraguay, Peru, and Uruguay. The survey distribution strategy for each country was developed by local partners with an intimate understanding of their country’s health care structure and physician workforce and included either distribution by an institutional leader to colleagues or via preexisting country listservs (eTable 1 in Supplement 1). These local leaders would identify potential participants and distribute the survey electronically via email or WhatsApp. Local leaders recorded the number of physicians who received the survey and sent a reminder email at least 1 week prior to the survey closing. The survey was distributed from August 1, 2020, through January 31, 2021, and was open at each site or country for 4 to 8 weeks. Only respondents who answered all questions regarding the ideal vs actual timing of PPC and the barriers for integration of PPC were included in this secondary analysis.

### Statistical Analysis

Country-specific demographic data were reported via descriptive statistics.^1,24^ For secondary analyses, the 5-point Likert scale was collapsed into 3 categories (often or always, sometimes, and never or rarely) to compare the associations of specific demographic variables by performing the Pearson χ^2^ test or the Fisher exact test. The McNemar test was used to assess responses regarding the actual vs ideal timing of PPC consultation. Analysis of variance was used to compare mean values for perceived barriers by income level. All *P* values were from 2-sided tests and results were deemed statistically significant at *P* < .05. All summaries and analyses were performed with SAS software, version 9.4 (SAS Institute Inc).^36^

#### Qualitative Analysis

Written, free-text responses for survey questions assessing differences between ideal and actual timing of PPC consultation and additional local barriers were translated into English by bilingual members of the study team (M.J.M. and S.R.G.). The qualitative codebook developed in previous ADAPT studies^29^ was used. Additional codes unique to this study were inductively identified through memo-writing of the free-text responses and included in analysis (see eTable 2 in Supplement 1 for final codebook). The updated codebook was subsequently piloted by 2 coders (M.J.M. and S.R.G.). Each free-text response served as the unit of analysis. After double coding the first 20% of responses, the coders achieved a κ value of 0.82, demonstrating excellent interrater reliability. Thematic content analysis was then performed to identify reasons for the difference between ideal and actual timing of PPC involvement, along with other common barriers to PPC integration for children with cancer. The qualitative data were managed using MAXQDA 2020 software (MAXQDA).^37^

## Results

### Participant Demographic Characteristics

A total of 874 physicians participated in the ADAPT survey, and of these, 831 (95.1%; 578 women [69.6%] and 253 men [30.4%]; 275 physicians [33.1%] aged <35 years and 556 physicians [66.9%] aged ≥35 years) completed all the questions on timing and barriers and were included in this analysis (Table 1). The median country response rate was 51.4% (range, 22.2%-88.9%), and the overall response rate was 37.9% (831 of 2193) (eTable 1 in Supplement 1). Most respondents had worked for at least 11 years (455 [54.8%]) (Table 1). General pediatrics was the most common specialty (268 [32.3%]), and an additional 241 physicians (29.0%) were pediatric hematologist-oncologists. Most respondents had no previous palliative care training (450 [54.2%]), and 275 (33.1%) did not have access to palliative care consultation in their institution. Of those who reported access to palliative care consultation (556 [66.9%]), nearly all (539 [96.9%]) reported access to a physician with less frequent access to other multidisciplinary team members (psychologist, 144 of 556 [25.9%]; nurse, 111 of 556 [20.0%]; and social worker, 67 of 556 [12.1%]). Most respondents (741 [89.2%]) had at least 1 patient die while in their care in the previous year.

**Table 1.** Participant Demographic Characteristics

Characteristic	Overall sample, No. (%) (N = 831)
Country	
Argentina	59 (7.1)
Bolivia	25 (3.0)
Chile	57 (6.9)
Colombia	96 (11.6)
Costa Rica	12 (1.4)
Dominican Republic	48 (5.8)
Ecuador	20 (2.4)
El Salvador	18 (2.2)
Guatemala	18 (2.2)
Haiti	9 (1.1)
Honduras	42 (5.1)
Mexico	188 (22.6)
Nicaragua	4 (0.5)
Panama	32 (3.9)
Paraguay	138 (16.6)
Peru	49 (5.9)
Uruguay	16 (1.9)
Age, y	
<35	275 (33.1)
≥35	556 (66.9)
Gender	
Female	578 (69.6)
Male	253 (30.4)
Primary medical specialty	
General pediatrician	268 (32.3)
Pediatric hematology and/or oncology	241 (29.0)
Pediatric palliative care	35 (4.2)
Other specialties^a^	287 (34.5)
Primary institution	
General hospital	368 (44.3)
Children’s hospital	326 (39.2)
Cancer hospital	113 (13.6)
Other	24 (2.9)
Years of experience	
≤10	376 (45.2)
≥11	455 (54.8)
Training palliative care	
Yes	381 (45.8)
No	450 (54.2)
Access to palliative care consultation in primary institution	
Yes	556 (66.9)
No	275 (33.1)
No. of patients who died in previous year	
0	90 (10.8)
1-5	439 (52.8)
≥6	302 (36.3)

^a^ Other specialties: pediatric anesthesiology, pediatric surgery, pediatric intensive care, adult palliative care, general internal medicine and/or family medicine, adult hematology and/or oncology, adult anesthesiology, adult surgery, adult intensive care, pediatric infectious diseases, pediatric subspecialty, surgical subspecialty, and other subspecialty.

### Timing of Palliative Care

**Figure 1.**
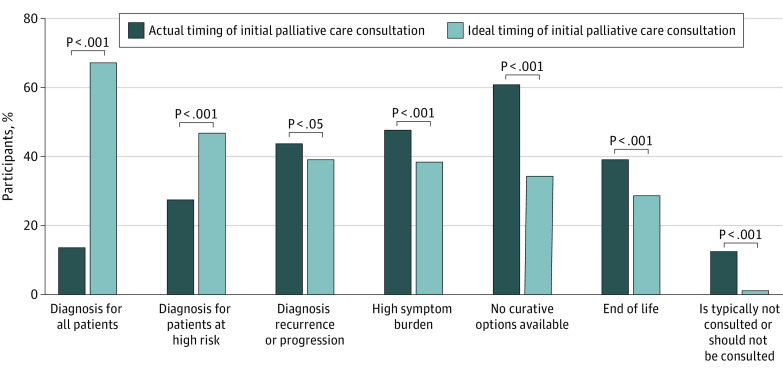
Ideal vs Actual Timing of Initial Palliative Care Consultation Results of 831 physician respondents to the multiple-choice questions asking when an initial palliative care consultation for a child with cancer typically occurs in their practice setting and when they believe is the ideal time it should occur. Participants were able to choose all options that were applicable.

Most participants (516 [62.1%]) stated that the actual timing of consultation about PPC for children with cancer in their center occurs when there are no further curative treatment options (Figure 1). Other common times for consultation included when patients have complex or high symptom burdens (405 [48.7%]), at times of disease relapse or progression (370 [44.5%]), or at the end of life (331 [39.8%]). A total of 106 respondents (12.8%) stated that the palliative care team was never consulted due to unavailability. Physicians working at a general hospital were more likely than those working at a children’s hospital or cancer hospital to state that PPC is not typically consulted for children with cancer because it is not available (general hospital, 65 of 350 [18.6%]; children’s hospital, 26 of 323 [8.0%]; cancer hospital 6, of 107 [5.6%]; *P* < .001) (eTable 3 in Supplement 1).

When asked to indicate the ideal timing for PPC consultation, most participants (572 [68.8%]) stated that this involvement should occur at diagnosis for all children with cancer, but only 117 (14.1%) stated that this happens in their actual practice (*P* < .001). In addition, 399 (48.0%) stated that palliative care professionals should be involved at diagnosis for patients at high risk, but this occurred for only 235 respondents (28.3%) (*P* < .001). A total of 355 respondents (42.7%) said that palliative care consultation often or always occurred too late in the treatment of a child with cancer. Very few respondents (10 [1.2%]) mentioned that palliative care should never be consulted even if available. There were no statistically significant differences in responses to ideal timing of care by the physician’s primary institution.

### Barriers

All barriers assessed in the survey, except for language (403 [48.5%]), were identified by at least half of the respondents as somewhat or extremely important to impeding early integration of palliative care for children with cancer (Table 2). The barriers most frequently identified as important included lack of home-based services (713 [85.8%]), limited physician knowledge on the role of palliative care (693 [83.4%]), physician discomfort in raising the topic of palliative care with families (676 [81.3%]), limited access to palliative care specialists or services (654 [78.7%]), and family resistance to involvement of palliative care (603 [72.6%]).

**Table 2.** Barriers to the Early Integration of Palliative Care

Barrier	Respondents indicating somewhat and/or extreme importance, No. (%) (N = 831)	Mean (SD) score^a^
Limited physician knowledge on the role of palliative care	693 (83.4)	3.99 (0.97)
Physician discomfort in raising the topic of palliative care with families	676 (81.3)	3.89 (0.92)
Physician desire to maintain hope	567 (68.2)	3.66 (0.98)
Uncertainty about patient prognosis	504 (60.6)	3.47 (1.02)
Family resistance to involvement of palliative care	603 (72.6)	3.79 (0.99)
Time constraints of pediatric oncologists during consultation	493 (59.3)	3.50 (1.16)
Lack of home-based services	713 (85.8)	4.19 (1.00)
Limited access to opioids	482 (58.0)	3.41 (1.25)
Limited access to palliative care specialists or services	654 (78.7)	3.97 (1.12)
Cost of palliative care consultation and treatment	445 (53.5)	3.31 (1.25)
Cultural differences between patients or families and physicians	537 (64.6)	3.60 (1.12)
Language differences between patients or families and physicians	403 (48.5)	3.19 (1.26)

^a^ The 5-point Likert scale was assigned numeric values (with 1 indicating “extremely unimportant” and 5 indicating “extremely important”); the mean score was calculated for each barrier on the basis of the numeric value assigned by all respondents.

Physician specialty and previous palliative care training were significantly associated with the variation in perceived importance of specific barriers. Pediatric palliative care clinicians were less likely than general pediatricians, pediatric hematologists-oncologists, and physicians from other specialties to find family resistance (PPC physicians, 13 of 35 [37.1%]; general pediatricians, 208 of 268 [77.6%]; pediatric hematologists-oncologists, 176 of 241 [73.0%]; physicians from other specialties, 206 of 287 [71.8%]; *P* < .001) or cost of palliative care (PPC physicians, 9 of 35 [25.7%]; general pediatricians, 169 of 268 [63.1%]; pediatric hematologists-oncologists, 96 of 241 [39.8%]; physicians from other specialties, 171 of 287 [59.6%]; *P* < .001) as important barriers (eTable 4 in Supplement 1). Physicians with previous palliative care training were less likely than those without previous palliative care training to identify limited access to opioids (209 of 381 [54.9%] vs 273 of 450 [60.7%]; *P* = .002), costs of palliative care treatment (172 of 381 [45.1%] vs 273 of 450 [60.7%]; *P* < .001), cultural differences between patients or families and the medical team (220 of 381 [57.7%] vs 317 of 450 [70.4%]; *P* < .001), and differences in language (165 of 381 [43.3%] vs 238 of 450 [52.9%]; *P* = .02) as important barriers in the early integration of palliative care for children with cancer (eTable 5 in Supplement 1).

**Figure 2.**
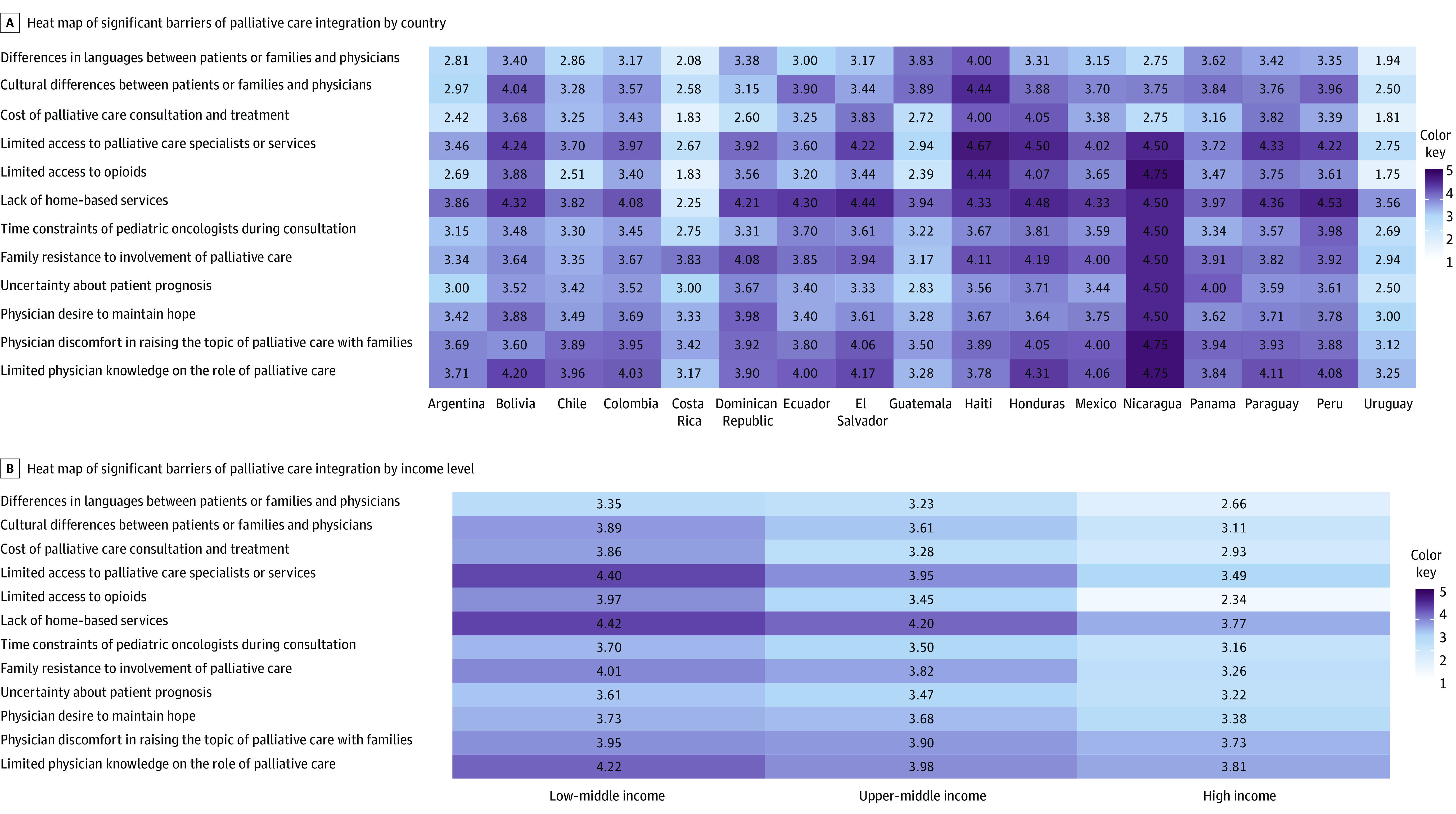
Mean Score of Significant Barriers to Palliative Care Integration by Country and Income Level A, The heat map shows, by country, each potential barrier to the early integration of palliative care for children with cancer as well as barriers’ perceived importance as identified by the participants (n = 831). The 5-point Likert scales were converted to numeric values with 1 indicating extremely unimportant and 5 indicating extremely important barriers. The numbers represented in the figure demonstrate the mean score within each country for each barrier. The darker the color, the higher the numeric value. B, The heat map shows, by World Bank income level, each potential barrier to the early integration of palliative care for children with cancer as well as the barriers’ perceived importance as identified by the participants (n = 831). The responses are shown as either low-middle income, upper-middle income, or high income. The 5-point Likert scales were converted to numeric values, with 1 indicating extremely unimportant and 5 indicating extremely important barriers. The numbers represented in the figure demonstrate the mean score within each income level for each barrier. The darker the color, the higher the numeric value.

Barriers also varied in importance based on country and country income level (Figure 2A and B). The mean score of every barrier included in the survey was highest for the LMICs, in the middle range for the upper-middle income countries (UMICs), and lowest for the HICs (Table 3). The most pronounced difference occurred for limited access to opioids (mean (SD) score: 3.97 [0.96] in LMICs, 3.45 [1.22] in UMICs, and 2.34 [1.24] in HICs; *P* < .001; Likert scale 1-5, with 1 being unimportant and 5 being very important) (Figure 2B).

**Table 3.** Mean Scores by World Bank Status

Variable	Score, mean (SD)			*P* value^a^
	Low-middle income (n = 98)	Upper-middle income (n = 660)	High income (n = 73)	
Limited physician knowledge on the role of palliative care	4.22 (0.79)	3.98 (0.99)	3.81 (0.98)	.01
Physician discomfort in raising the topic of palliative care with families	3.95 (0.92)	3.90 (0.91)	3.73 (1.02)	.24
Physician desire to maintain hope	3.73 (0.89)	3.68 (0.98)	3.38 (1.06)	.03
Uncertainty about patient prognosis	3.61 (1.01)	3.47 (1.02)	3.22 (0.98)	.04
Family resistance to involvement of palliative care	4.01 (0.92)	3.82 (0.97)	3.26 (1.07)	<.001
Time constraints of pediatric oncologists during consultation	3.70 (1.08)	3.50 (1.17)	3.16 (1.17)	.01
Lack of home-based services	4.42 (0.73)	4.20 (1.00)	3.77 (1.17)	<.001
Limited access to opioids	3.97 (0.96)	3.45 (1.22)	2.34 (1.24)	<.001
Limited access to palliative care specialists or services	4.40 (0.83)	3.95 (1.11)	3.49 (1.30)	<.001
Cost of palliative care consultation and treatment	3.86 (1.12)	3.28 (1.23)	2.93 (1.37)	<.001
Cultural differences between patients or families and physicians	3.89 (1.09)	3.61 (1.10)	3.11 (1.12)	<.001
Differences in languages between patients or families and physicians	3.35 (1.33)	3.23 (1.23)	2.66 (1.33)	<.001

^a^ Analysis of variance test.

When asked to explain the difference between actual vs ideal timing of palliative care consultation in their setting, respondents described a lack of access, knowledge, and adequate staffing. One respondent stated, “I believe that the main impediment is the lack of knowledge, the oncologists think that palliative care means that there is nothing to [do] anymore and that only children that are dying should receive it.” Another respondent described limited staffing impeding early integration: “In my hospital, the palliative care service sees pediatric and adult patients, and they are overwhelmed with work, so it is not our practice that all patients at diagnosis are known to the group as it would saturate the service.”

Other respondents described common misconceptions surrounding palliative care, including that it addressed only physical symptoms, was associated only with end-of-life care, or that it would damage the relationship between the patient or family and oncologist. One respondent described the experience at their own institution by saying, “Many colleagues fear involving the palliative care team because they believe they will have a negative connotation for the family, as there is still some perception that palliative care should be initiated when there is no longer a curative option, and consultation is usually done late with the team.”

## Discussion

Despite the WHO’s recommendations for early integration of PPC in the care of children with cancer, barriers impeding this integration remain globally. This ADAPT study provides a comprehensive assessment of barriers to PPC integration in cancer care within 17 countries in Latin America. Findings demonstrate that physicians in the region identify the ideal timing of PPC involvement to be earlier in the treatment of children with cancer compared with the actual timing of PPC consultation in their setting, which typically occurs at the end of life or when no further curative options are available. In addition, respondents highlighted several barriers to integration of PPC, including limited capacity for home-based services, limited access to PPC support, limited physician knowledge and physician discomfort in discussing PPC, and concerns about familial resistance to PPC involvement. These findings underscore the variation in barriers between countries with different resources and infrastructure, as participants from LMICs rated all barriers as more significant than participants from HICs.

Overall, these findings from Latin America are similar to those from the ADAPT study in Eurasia.^28^ Specifically, physicians in both Latin America and Eurasia highlighted the importance of earlier integration of PPC for children with cancer than commonly occurs in their setting. In addition, despite differences in language, culture, and health care infrastructure, the top 5 identified barriers in Eurasia were the same as in Latin America. These barriers included limited access to home-based services, physician PPC knowledge, access to PPC specialists, perceived family resistance, and physician discomfort.^28^ These findings highlight global challenges to PPC integration in childhood cancer care, representing potential areas for future interventions, including clinical interventions such as the development of home-based services along with educational initiatives to improve physician and family knowledge. However, such interventions must be implemented and adapted to the context of each country’s unique setting and challenges.

In addition, previous PPC training and physician specialty resulted in different responses. For example, physicians with PPC training did not feel that lack of access to opioids was as high of a barrier as those without previous PPC training. This finding may demonstrate the importance of PPC training to provide education on the range of pharmacologic and nonpharmacologic approaches to the management of pain and other symptoms. This finding was seen in previously published data from this survey demonstrating higher comfort in addressing physical symptoms of patients, including pain, despite not having access to certain medications.^25^ Also, PPC physicians felt that family resistance was not as important as other barriers. This finding may demonstrate that the barrier of family resistance is more of a resistance from the physicians themselves than from the family. Research in HICs has demonstrated that families are open to PPC involvement from diagnosis.^38^ Future research is needed to assess parent and family perceptions of PPC in LMICs.

One key difference between the 2 regions was access to palliative care consultation, which was higher in Latin America than Eurasia (66.9% [556 of 831] vs 54.2% [230 of 424]).^27^ Many respondents from Latin America underscored that palliative care teams exist but are functionally unavailable due to limited staffing and high workloads that prohibit earlier consultation. These challenges also exist among HICs; a recent systematic review of mostly HIC studies showed that only 54.5% of pediatric oncology patients received PPC before death, and most services occurred when no further curative options were available or at the end of life.^39^ This lack of PPC involvement or this delayed timing of PPC consultation was found to be associated with limited staffing of health care professionals trained in PPC.^17,40,41^ Facilitation of education and training to increase PPC services is necessary but not sufficient to improve access; it is also essential to build capacity and increase the palliative care workforce to promote earlier involvement in patient care.

The combination of education of physicians who care for children with cancer along with the building of the palliative care workforce will provide opportunities to bridge the gap between the ideal and actual timing and ensure pediactric patients with cancer receive the benefit associated with earlier integration of PPC. For example, the most common specialty in this cohort was pediatricians. For many institutions, general pediatricians provide direct patient care for children with cancer under the supervision of a pediatric hematology-oncology consultant. Targeted educational interventions teaching general pediatricians the principles of primary palliative care would increase the knowledge among the physician population most likely to provide care for children with cancer. This intervention ensures that the patients’ symptoms are still being managed while working to build capacity in subspecialty care.^20^

Finally, although many similarities exist among countries and regions, differences remain, especially with regard to available resources. In Latin America, LMICs had higher mean scores than HICs in every barrier evaluated. This finding is particularly relevant when considering access to essential medicines in PPC, such as opioids, because this access is more limited in LMICs than in UMICs and HICs.^17,41^ Despite a higher need for PPC in LMICs due to higher childhood cancer mortality, the lower availability of and barriers to delivery of PPC widen existing global disparities in quality of care. Although opportunities for synergy between regions and countries exist, it is critical that capacity-building efforts focus on equitable access to basic resources and support for global PPC between countries.

### Limitations

This study has several limitations. First, the survey was distributed in Spanish, which precluded certain countries throughout Latin America in which Spanish is not the primary language. However, future efforts should include translation to Portuguese and distribution in Brazil. Also, because of differences in physician workforce and health care infrastructure between countries, specific distribution strategies tailored to each country’s resources and needs were used. These different strategies resulted in varying response rates and, in some countries, having a sample size disproportionate to their physician population. This study’s large sample size, however, increases confidence that the study is representative of existing barriers through the quantitative and qualitative responses. In addition, physician specialty and training were associated with their responses; therefore, we were intentional in including any physician who cared for children with cancer. The final sample included participants representing more than 30 physician subspecialties, ensuring as many voices from different specialties as possible. Also, this survey was distributed electronically, which may have excluded physicians caring for children in more rural settings with less access to technology and the internet. However, childhood cancer care in Latin America typically occurs in large urban centers, and this study is likely representative of physicians responsible for most pediatric oncology care in the region. This study did not explore the characteristics of hospitals in which these physicians practiced, such as funding mechanisms and resources. In future work, we will explore the hospital characteristics and their potential association with PPC provision. The survey also does not always differentiate between primary and specialty palliative care, which may have altered responses and may lead to different solutions to address the barriers to early integration. Finally, this survey was distributed among physicians and therefore lacks multidisciplinary perspectives. Future efforts should assess the perceptions of other members of the interdisciplinary team.

## Conclusions

This survey study highlights the discrepancy between ideal and actual timing of PPC involvement for children with cancer and the barriers to PPC integration in Latin America. Physicians in Latin America believe that PPC should be integrated early in childhood cancer care, yet they recognize that this early involvement rarely happens in their settings. Barriers to earlier involvement of PPC include lack of resources, personnel, and knowledge of palliative care, along with physicians’ beliefs that there is resistance to PPC by other physicians and patients’ families. Although there are common challenges among countries within Latin America, interventions should be contextually adjusted for each country’s unique setting and equitably distributed to reduce, rather than widen, existing disparities. Concerted efforts in education, advocacy, and capacity building focused on addressing these barriers may help improve access to high-quality PPC for children with cancer in the region.

## Notes

Supplement 1. **eAppendix.** ADAPT LA Survey **eTable 1.** Country-Specific Distribution Method **eTable 2.** ADAPT LA Qualitative Codebook on Barriers **eTable 3.** Actual vs Ideal Timing by Primary Institution **eTable 4.** Important Barriers by Specialty **eTable 5.** Important Barriers by Previous Palliative Care Training

Supplement 2.
Nonauthor Collaborators

Supplement 3.
Data Sharing Statement
