# Supplementary material for: Ideal vs Actual Timing of Palliative Care Integration for Children With Cancer in Latin America
Source: JAMA Netw Open. 2023 Jan 19;6(1):e2251496. doi: 10.1001/jamanetworkopen.2022.51496 (PMC9857245; doi:10.1001/jamanetworkopen.2022.51496)
Supplement: Supplement 1. — eAppendix. ADAPT LA Survey eTable 1. Country-Specific Distribution Method eTable 2. ADAPT LA Qualitative Codebook on Barriers eTable 3. Actual vs Ideal Timing by Primary Institution eTable 4. Important Barriers by Specialty eTable 5. Important Barriers by Previous Palliative Care Training [file jamanetwopen-e2251496-s001.pdf]

Supplementary Online Content

McNeil MJ, Ehrlich B, Wang H, et al; Assessing Doctors' Attitudes on Palliative Treatment (ADAPT) Latin America Study Group. Ideal vs actual timing of palliative care integration for children with cancer in Latin America. *JAMA Netw Open.* 2023;6(1):e2251496. doi:10.1001/jamanetworkopen.2022.51496

eAppendix. ADAPT LA Survey
eTable 1. Country-Specific Distribution Method
eTable 2. ADAPT LA Qualitative Codebook on Barriers
eTable 3. Actual vs Ideal Timing by Primary Institution
eTable 4. Important Barriers by Specialty
eTable 5. Important Barriers by Previous Palliative Care Training

This supplementary material has been provided by the authors to give readers additional information about their work.

© 2023 McNeil MJ et al. *JAMA Network Open.*

eAppendix. ADAPT LA Survey

Demographics

1.“Do you treat children with cancer as part of your medical practice?” (Yes/no)

2. What is the country in which you currently practice medicine?

Argentina
Bolivia
Brazil
Chile
Colombia
Costa Rica
Dominical Republic
Ecuador
El Salvador
Guatemala
Haiti
Honduras
Mexico
Nicaragua
Panama
Paraguay
Peru
Uruguay

3. Your age:

<35 years
35 to 50 years
51 to 65 years
>65 years

4. Your gender:

Female
Male
Other
Prefer not to disclose

5. Would you consider yourself a religious person? (religious: the search for significance that occurs within the context of established faith traditions (Judaism, Christianity, Muslim, Hindu, Buddhism, etc.)

Strongly Disagree
Disagree
Uncertain
Agree
Strongly Agree

© 2023 McNeil MJ et al. *JAMA Network Open.*

6. Would you consider yourself a spiritual person? (spiritual: individual search for meaning and purpose)

- Strongly Disagree
- Disagree
- Uncertain
- Agree
- Strongly Agree

7. Please indicate your primary medical specialty:

- General pediatrician
- Pediatric hematology and/or oncology
- Pediatric anesthesiology
- Pediatric surgery
- Pediatric intensive care
- Pediatric Palliative care
- Adult Palliative Care
- General Internal Medicine/Family Medicine
- Adult hematology and/or oncology
- Adult anesthesiology
- Adult surgery
- Adult intensive care
- Other (describe)

8. How many years of experience in practice do you have since graduating medical school?

- 0-5 years
- 6-10 years
- 11-15 years
- 16-20 years
- 21+ years

9. Concerning the primary institution in which you work, indicate if it is a:

- General Hospital
- Children's Hospital
- Cancer Hospital
- Other (describe)

10. Have you received any formal training in palliative care?

- Yes
- No

If Yes (check all that apply):

- Continuing Medical Education (or postgraduate course without official certification)
- Certificate course
- Medical School or Post-graduate Rotation

© 2023 McNeil MJ et al. *JAMA Network Open.*

Undergraduate/ Medical School Course
Master's in Palliative Care
Residency or Fellowship in palliative care
Other: (Please describe)

11. Do you have access to a pediatric palliative care expert for consultation in your practice? (Yes/No)

If yes, please check the type(s) of palliative care experts available:

Physician, nurse, social worker, psychologist, other (please describe)

12. How many pediatric patients in your care (less than 18 years old) died in the last 12 months?

a. 0 patients
b. 1-5 patients
c. 6-10 patients
d. 11-20 patients
e. 21+ patients

13. What does palliative care mean to you?

Perspectives

Please rate the extent to which you agree with the following statements about ***pediatric oncology patients*** *receiving* ***palliative care****. Please check one box per line:*

	Strongly Disagree	***Somewhat Disagree***	***Neutral***	***Somewhat Agree***	***Strongly Agree***

14. It is difficult to know when a patient with cancer would most benefit from meeting the palliative care team.

15. Quality of life is often overlooked in the face of cancer-directed treatment.

16. Children with advanced and incurable cancer often suffer at the end-of-life.

17. Early consultation with palliative care causes increased parental burden and anxiety.

18. Palliative care is perceived by parents as meaning the end of life is near and that the oncologist will 'give up' on their child.

19. Palliative care can be integrated with disease-directed therapy.

20. Involving palliative care suggests the oncologist has failed in the mission to cure the patient.

© 2023 McNeil MJ et al. *JAMA Network Open.*

21. There are situations where it is in a dying child's best interest to remove mechanical ventilation if in alignment with the family's wishes.

22. Palliative care for children with cancer can be delivered by health care workers of all disciplines, not only by palliative care specialists.

23. Palliative care is synonymous with "end-of-life" care.

24. Involvement of palliative care during cancer therapy gives greater attention to quality of life and symptom management (e.g. pain, constipation, dyspnea, fatigue).

25. Involvement of palliative care undermines the role of the pediatric oncologist as the physician in charge of patient care.

26. Children with cancer who receive palliative care die earlier than those who do not.

27. Early integration of palliative care for all children diagnosed with cancer would decrease patient suffering.

28. Early integration of pediatric palliative care with cancer care would improve interdisciplinary communication.

29. Palliative care is incompatible with curative care.

30. Involving the palliative care team early has negative effects on the relationship between the oncologist and the patient and family.

31. Palliative care is appropriate at any stage of treatment in a child with high-risk cancer.

32. Administering opioids to patients in pain hastens death due to respiratory depression.

33. In my setting, physicians typically continue to recommend cancer-directed treatment for children with incurable oncological disease even when that treatment is ineffective or unlikely to prolong a child's life.

34. In my setting, doctors generally feel confident taking care of the physical needs of pediatric patients with serious incurable illness.

35. In my setting, doctors generally feel confident taking care of the emotional needs of pediatric patients with serious incurable illness.

36. In my setting, doctors generally feel confident taking care of the spiritual needs of pediatric patients and their families with serious incurable illness.

© 2023 McNeil MJ et al. *JAMA Network Open.*

37. In my setting, doctors generally feel confident providing grief and bereavement care to the families of children who die.

38. Greater emphasis on palliative care education for doctors and health care professionals is an important step in improving access to palliative care.

39. I wish to have more education on how to provide palliative care to my patients.

Individual Experience:
Please rate the ***frequency*** *of the following statements about* ***your individual experiences*** *with* ***pediatric oncology patients****. Please check one box per line:*

	Never	***Rarely***	***Sometimes***	***Often***	***Always***

40. In my setting of practice, palliative care consultation is available when I feel it is needed for a child with cancer.

41. In my setting, I have felt that involvement of palliative care has occurred too late in the treatment of a child with cancer.

42. In my setting, I have acted against my conscience by providing **aggressive treatment** to a pediatric oncology patient with advancing disease.

43. I feel confident assessing and treating the **physical needs** of pediatric patients with serious incurable illness.

44. I feel confident assessing and treating the **emotional needs** of pediatric patients with serious incurable illness and their families.

45. I feel confident taking care of the **spiritual needs** of pediatric patients and their families with serious incurable illness.

46. I feel confident providing grief and bereavement care to the families of children who die.

47. I have felt burdened by my inability to control the suffering of children at the end-of-life.

Multiple choice:
Please choose ***all*** *that apply for every option.*

48. The role of palliative care in the care of children with cancer is (please choose all that apply):

- To aid in reducing pain and suffering related to disease and/or treatment
- To provide psychological support to the patient and their family
- To provide spiritual support to the patient and their family
- To aid in family decision-making around treatment options

- To help clarify the goals of care of the patient and family
- To help communicate bad news to patients and families
- To aid in communication between the patient, family, and medical teams
- To assist with transitions from the hospital to hospice or home at end-of-life
- Other: Please describe

49. When does **initial** palliative care consultation for a child with cancer **typically** occur in your setting (please choose all that apply):

- At the time of cancer diagnosis for all patients
- At the time of cancer diagnosis for patients at high-risk of relapse or progression
- At the time of disease relapse or progression
- At the time of complex or high symptom burden (pain, suffering)
- When there are no longer curative therapeutic options available
- At the end of life
- Palliative care is typically not consulted for children with cancer (because it is not necessary or not available)

50. Assuming unlimited resources, when do you think is the **ideal** timing of **initial** palliative care consultation for a child with cancer (please choose all that apply):

- At the time of cancer diagnosis for all patients
- At the time of cancer diagnosis for patients at high-risk of relapse or progression
- At the time of disease relapse or progression
- At the time of complex or high symptom burden (pain, suffering)
- When there are no longer curative therapeutic options available
- At the end of life
- Palliative care consultation is never necessary in pediatric cancer care

51. If there is a difference between when initial palliative care consultation **typically occurs** in your setting and what you think is **ideal**, why do you think this difference exists?

Barriers*:*
Please rate the extent to which you feel the following statements represent barriers to ***early integration of palliative care for pediatric oncology patients****. Please check one box per line:*

	Extremely Unimportant Barrier	***Somewhat Unimportant Barrier***	***Neither Important nor Unimportant Barrier***	***Somewhat Important Barrier***	***Extremely Important Barrier***

52. Limited physician knowledge on the role of palliative care

53. Physician discomfort in raising the topic of palliative care with families

54. Physician desire to maintain hope

55. Uncertainty about patient prognosis

56. Family resistance to involvement of palliative care

© 2023 McNeil MJ et al. *JAMA Network Open.*

57. Time constraints of pediatric oncologists during consultation

58. Lack of home-based services

59. Limited access to opioids

60. Limited access to palliative care specialists or services

61. Cost of palliative care consultation and treatment

62. Cultural differences between patients/families and physicians

63. Differences in languages between patients/families and physicians

64. Are there barriers to early integration of palliative care for pediatric oncology patients not listed above? If yes, please identify the barriers and rate their importance below. If no additional barriers exist, go to the next question.

65. Do you have any additional comments or concerns regarding palliative care for children and adolescents with cancer?

© 2023 McNeil MJ et al. *JAMA Network Open.*

eTable 1. Country-Specific Distribution Method

**Country**	**Distribution Method**	**No. of surveys distributed**	**No. of surveys completed**	**Response Rate, %**
**Argentina**	Distribution by formalized country contact list	266	59	22.2%
**Bolivia**	Distribution by institution contact list	52	25	48.1%
**Chile**	Distribution by institution contact list	184	57	31.0%
**Colombia**	Distribution by institution contact list	321	96	29.9%
**Costa Rica**	Distribution by institution contact list	42	12	28.6%
**Dominican Republic**	Distribution by institution contact list	55	48	87.3%
**Ecuador**	Distribution by institution contact list	37	20	54.1%
**El Salvador**	Distribution by institution contact list	22	18	81.8%
**Guatemala**	Distribution by institution contact list	35	18	54.3%
**Haiti**	Distribution by formalized country contact list	21	11	51.4%
**Honduras**	Distribution by institution contact list	52	42	80.8%
**Mexico**	Distribution by institution contact list	585	188	32.1%
**Nicaragua**	Distribution by institution contact list	6	4	66.7%
**Panama**	Distribution by institution contact list	75	32	42.7%
**Paraguay**	Distribution by formalized country contact list	339	159	40.7%
**Peru**	Distribution by institution contact list	83	49	59.0%
**Uruguay**	Distribution by institution contact list	18	16	88.9%
**Total**		2,193	831	37.9%
**Median Response Rate**				51.4%

© 2023 McNeil MJ et al. *JAMA Network Open.*

eTable 2. ADAPT LA Qualitative Codebook on Barriers

Category	Code	Definition
Actions/ Desires		
	Satisfied with Status Quo	Refers to any opinions that indicate that the status quo is currently satisfactory or that palliative care should not exist.
	Need for systemic change	References to desires to integrate or develop higher quality or improvement of palliative care services in the future (e.g. provision of "individualized care based on need"). This refers to provider perspectives of what should be, or what they hope should happen in the future, and who "should" be responsible for palliative care, if this implies training more palliative care specialists. Note: this is different than a right to have palliative care (coded as "A right" in positive attitudes), and not all statements using "should" indicate desire for change, but rather a factual statement.
Barriers		
	Physician Attitudes	Refers to any physician attitudes that serve as a barrier to early integration of palliative care. Includes provider lack of education, awareness about palliative care, or hesitation to administer drugs (pain medication). This also includes provider feelings of ambition, failure, hope, or burnout that prevent earlier referral, and perceptions that there are no opportunities to include other specialists or other challenges with interdisciplinary communication. It also includes provider choices to not communicate diagnoses or prognoses with the patient/family, but does NOT include provider perceptions of parent un-readiness (code this as "family attitudes"). It also does NOT include health systems barriers such as lack of drug access (code as "systemic") or lack of palliative care experts (code as "access"). Note, this includes mentions of education pertaining to specific individuals.
	Family Attitudes	Refers to family attitudes which serve as a barrier to early integration of palliative care. Includes mentions of family lack of education, misperceptions, or awareness. This further includes family readiness or hope that leads to refusal of palliative care. Includes mentions of difficulty communicating with the family, as well as family not communicating with their child.
	Society Attitudes	Any references to the attitudes of the greater community or society which serve as a barrier to early integration of palliative care. Includes mentions society misperceptions, stigma, or greater "culture".
	Institutional	This code includes any structural barriers within the institution (hospital, organization, center) that prevent early integration of palliative care, but NOT on a larger legislative/federal health systems level (e.g. hospital collaboration, bed shortage, infrastructural limits within the hospital). This further includes understaffing, heavy oncologist workload, lack of administrative support, or administrative ignorance/lack of education.
	Systemic	This refers to larger systemic/federal level (health systems) barriers that prevent early integration of palliative care. This includes lack of organized education (on a health systems level without mention to specific individuals, e.g. "the opportunity to specialize"), poor legislation, lack of accepted standards, lack of funding, strict drug regulations, lack of drug access/procurement, or poor infrastructure/distribution of resources across

© 2023 McNeil MJ et al. *JAMA Network Open.*

		regions. Includes any mention of laws or policies. This also includes general mention of resources (e.g. financial). Note, the focus is not on individual lack of training or choices to not administer drugs (code as “physician attitudes”).
	Access	Refers to access to <palliative care> specialists, services, and consults that serve as a barrier to its integration, including existence or access to a trained specialist, or a palliative care service/team (e.g. "not available care", "not enough doctors"). Does NOT include reference of access to overworked general providers/oncologists (code as “institutional”). E.g. "need to develop palliative care in all areas".
Timing of Palliative Care		
	At diagnosis	This references the timing of palliative care involvement at the time of diagnosis. Refers to a specific time point when palliative care is integrated. This does NOT imply exclusivity with curative treatment.
	Disease Progression	Includes references to any mention of specific time points of palliative care integration or involvement that are not at diagnosis, at the end-of-life, or after treatment options are exhausted. This includes involvement given poor prognosis for high-risk patients, disease relapse or progression, and high symptom burden (pain, suffering). This does not imply exclusivity with curative treatment. Does NOT include referencing palliative care using an individualized approach or an on-need basis, unless there are further specifications about timing in the course of treatment/life.
	Integration with Treatment	Any references to palliative care to be integrated concurrently with treatment, with NO specific mention a time point, such as at diagnosis (code as "At diagnosis") or disease relapse/high symptom burden (code as "Disease Progression"). Key phrases such as "early integration" included here. Note, this is mutually exclusive with other timing codes.
	No other options available	This references the timing of palliative care involvement as conditional upon patient unresponsiveness to treatment and/or other treatment options being unavailable and "exhausted" (e.g. "treatment is failing", "no options available") . Refers to a specific time point when palliative care is integrated or involved in a patient's disease course. Often there is an implication of mutual exclusivity between curative therapy and symptomatic treatment/palliative care. DO include any mention of "incurable" disease/patients under this code.
	End-of-life/Terminal Stages	Includes any references to the specific time points of palliative care integration or involvement at the end-of-life of a patient. This also includes grief and bereavement support after death. Consider this code when seeing terms such as a "last resort", "last days of life", or "final landing". Any reference to care delivered to temrinal patients, with no indication of previous exposure, is coded (e.g. "to improve quality of life of end-stage patients"). However, do NOT code words "until end-of-life" as it leaves open-ended when palliative care was initiated (code "end-of-life care" under component of palliative care). This will often be double-coded with "end-of-life care".

© 2023 McNeil MJ et al. *JAMA Network Open.*

eTable 3. Actual vs Ideal Timing by Primary Institution

	Primary institution				
	General Hospital (N=350)	*Children's Hospital (N=323)*	*Cancer Hospital (N=107)*	*Other (N=51)*	*P-values*
**When does initial palliative care consultation for a child with cancer typically occur in your setting (please choose all that apply)**					
At the time of cancer diagnosis for all patients	49 (14.0)	46 (14.2)	11 (10.3)	11 (21.6)	0.3018[a]
At the time of cancer diagnosis for patients at high-risk of relapse or progression	74 (21.1)	106 (32.8)	38 (35.5)	17 (33.3)	0.0015[a]
At the time of disease relapse or progression	116 (33.1)	171 (52.9)	60 (56.1)	23 (45.1)	<.0001[a]
At the time of complex or high symptom burden (pain, suffering)	153 (43.7)	165 (51.1)	60 (56.1)	27 (52.9)	0.0747[a]
When there are no longer curative therapeutic options available	201 (57.4)	207 (64.1)	79 (73.8)	29 (56.9)	0.0139[a]
At the end of life	128 (36.6)	129 (39.9)	51 (47.7)	23 (45.1)	0.1806[a]
Palliative care is typically not consulted for children with cancer (because it is not necessary or not available)	65 (18.6)	26 (8.0)	6 (5.6)	9 (17.6)	<.0001[a]
**Assuming unlimited resources, when do you think is the ideal timing of initial palliative care consultation for a child with cancer (please choose all that apply)**					
At the time of cancer diagnosis for all patients	236 (67.4)	225 (69.7)	73 (68.2)	38 (74.5)	0.7508[a]
At the time of cancer diagnosis for patients at high-risk of relapse or progression	156 (44.6)	161 (49.8)	53 (49.5)	29 (56.9)	0.2845[a]

© 2023 McNeil MJ et al. *JAMA Network Open.*

At the time of disease relapse or progression	134 (38.3)	132 (40.9)	47 (43.9)	19 (37.3)	0.7111[a]
At the time of complex or high symptom burden (pain, suffering)	124 (35.4)	140 (43.3)	43 (40.2)	21 (41.2)	0.2117[a]
When there are no longer curative therapeutic options available	112 (32.0)	123 (38.1)	40 (37.4)	17 (33.3)	0.3842[a]
At the end of life	92 (26.3)	99 (30.7)	36 (33.6)	16 (31.4)	0.4069[a]
Palliative care consultation is never necessary in pediatric cancer care	5 (1.4)	3 (0.9)	0	2 (3.9)	0.1887[b]

© 2023 McNeil MJ et al. *JAMA Network Open.*

eTable 4. Important Barriers by Specialty

	Specialty				
**Barrier**	**Pediatric Palliative Care (N=35)**	**General Pediatrician (N=268)**	**Pediatric Hematology and/or Oncology (N=241)**	**Other Specialty (N=287)**	**P-value**
**Limited physician knowledge on the role of palliative care**					
Unimportant Barrier	5 (14.3)	21 (7.8)	29 (12.0)	25 (8.7)	0.5403[b]
Neither Important nor Unimportant Barrier	2 (5.7)	20 (7.5)	19 (7.9)	17 (5.9)	
Important Barrier	28 (80.0)	227 (84.7)	193 (80.1)	245 (85.4)	
**Physician discomfort in raising the topic of palliative care with families**					
Unimportant Barrier	2 (5.7)	21 (7.8)	32 (13.3)	20 (7.0)	0.2294[b]
Neither Important nor Unimportant Barrier	2 (5.7)	29 (10.8)	20 (8.3)	29 (10.1)	
Important Barrier	31 (88.6)	218 (81.3)	189 (78.4)	238 (82.9)	
**Physician desire to maintain hope**					
Unimportant Barrier	4 (11.4)	41 (15.3)	40 (16.6)	38 (13.2)	0.8095[b]
Neither Important nor Unimportant Barrier	4 (11.4)	46 (17.2)	37 (15.4)	54 (18.8)	
Important Barrier	27 (77.1)	181 (67.5)	164 (68.0)	195 (67.9)	
**Uncertainty about patient prognosis**					
Unimportant Barrier	7 (20.0)	32 (11.9)	63 (26.1)	58 (20.2)	0.0017[b]
Neither Important nor Unimportant Barrier	5 (14.3)	55 (20.5)	53 (22.0)	54 (18.8)	
Important Barrier	23 (65.7)	181 (67.5)	125 (51.9)	175 (61.0)	
**Family resistance to involvement of palliative care**					
Unimportant Barrier	12 (34.3)	23 (8.6)	31 (12.9)	46 (16.0)	<.0001[a]
Neither Important nor Unimportant Barrier	10 (28.6)	37 (13.8)	34 (14.1)	35 (12.2)	
Important Barrier	13 (37.1)	208 (77.6)	176 (73.0)	206 (71.8)	
**Time constraints of pediatric oncologists during consultation**					
Unimportant Barrier	13 (37.1)	56 (20.9)	58 (24.1)	61 (21.3)	0.0538[a]
Neither Important nor Unimportant Barrier	9 (25.7)	53 (19.8)	32 (13.3)	56 (19.5)	
Important Barrier	13 (37.1)	159 (59.3)	151 (62.7)	170 (59.2)	

© 2023 McNeil MJ et al. *JAMA Network Open.*

**Lack of home-based services**					
Unimportant Barrier	4 (11.4)	25 (9.3)	20 (8.3)	22 (7.7)	0.0983[b]
Neither Important nor Unimportant Barrier	5 (14.3)	20 (7.5)	8 (3.3)	14 (4.9)	
Important Barrier	26 (74.3)	223 (83.2)	213 (88.4)	251 (87.5)	
**Limited access to opioids**					
Unimportant Barrier	10 (28.6)	80 (29.9)	71 (29.5)	60 (20.9)	0.1860[a]
Neither Important nor Unimportant Barrier	6 (17.1)	43 (16.0)	37 (15.4)	42 (14.6)	
Important Barrier	19 (54.3)	145 (54.1)	133 (55.2)	185 (64.5)	
**Limited access to palliative care specialists or services**					
Unimportant Barrier	7 (20.0)	37 (13.8)	43 (17.8)	28 (9.8)	0.1051[b]
Neither Important nor Unimportant Barrier	4 (11.4)	18 (6.7)	18 (7.5)	22 (7.7)	
Important Barrier	24 (68.6)	213 (79.5)	180 (74.7)	237 (82.6)	
**Cost of palliative care consultation and treatment**					
Unimportant Barrier	22 (62.9)	45 (16.8)	100 (41.5)	58 (20.2)	<.0001[b]
Neither Important nor Unimportant Barrier	4 (11.4)	54 (20.1)	45 (18.7)	58 (20.2)	
Important Barrier	9 (25.7)	169 (63.1)	96 (39.8)	171 (59.6)	
**Cultural differences between patients/families and physicians**					
Unimportant Barrier	12 (34.3)	46 (17.2)	54 (22.4)	48 (16.7)	0.0284[b]
Neither Important nor Unimportant Barrier	5 (14.3)	42 (15.7)	48 (19.9)	39 (13.6)	
Important Barrier	18 (51.4)	180 (67.2)	139 (57.7)	200 (69.7)	
**Language differences between patients/families and physicians**					
Unimportant Barrier	14 (40.0)	70 (26.1)	97 (40.2)	81 (28.2)	0.0007[a]
Neither Important nor Unimportant Barrier	11 (31.4)	47 (17.5)	46 (19.1)	62 (21.6)	
Important Barrier	10 (28.6)	151 (56.3)	98 (40.7)	144 (50.2)	

[a] Chi-Square Test; [b] Fisher's Exact Test

© 2023 McNeil MJ et al. *JAMA Network Open.*

eTable 5. Important Barriers by Previous Palliative Care Training

	Previous palliative care training		
**Barrier**	**Yes (N=381)**	**No (N=450)**	**P-value[a]**
**Limited physician knowledge on the role of palliative care**			
Unimportant Barrier	39 (10.2)	41 (9.1)	0.8495
Neither Important nor Unimportant Barrier	27 (7.1)	31 (6.9)	
Important Barrier	315 (82.7)	378 (84.0)	
**Physician discomfort in raising the topic of palliative care with families**			
Unimportant Barrier	37 (9.7)	38 (8.4)	0.8137
Neither Important nor Unimportant Barrier	36 (9.4)	44 (9.8)	
Important Barrier	308 (80.8)	368 (81.8)	
**Physician desire to maintain hope**			
Unimportant Barrier	63 (16.5)	60 (13.3)	0.2604
Neither Important nor Unimportant Barrier	58 (15.2)	83 (18.4)	
Important Barrier	260 (68.2)	307 (68.2)	
**Uncertainty about patient prognosis**			
Unimportant Barrier	85 (22.3)	75 (16.7)	0.1175
Neither Important nor Unimportant Barrier	75 (19.7)	92 (20.4)	
Important Barrier	221 (58.0)	283 (62.9)	
**Family resistance to involvement of palliative care**			
Unimportant Barrier	59 (15.5)	53 (11.8)	0.0544
Neither Important nor Unimportant Barrier	61 (16.0)	55 (12.2)	
Important Barrier	261 (68.5)	342 (76.0)	
**Time constraints of pediatric oncologists during consultation**			
Unimportant Barrier	95 (24.9)	93 (20.7)	0.1029
Neither Important nor Unimportant Barrier	75 (19.7)	75 (16.7)	
Important Barrier	211 (55.4)	282 (62.7)	
**Lack of home-based services**			
Unimportant Barrier	37 (9.7)	34 (7.6)	0.0686
Neither Important nor Unimportant Barrier	28 (7.3)	19 (4.2)	
Important Barrier	316 (82.9)	397 (88.2)	
**Limited access to opioids**			
Unimportant Barrier	123 (32.3)	98 (21.8)	0.0017
Neither Important nor Unimportant Barrier	49 (12.9)	79 (17.6)	
Important Barrier	209 (54.9)	273 (60.7)	

© 2023 McNeil MJ et al. *JAMA Network Open.*

**Limited access to palliative care specialists or services**			
Unimportant Barrier	64 (16.8)	51 (11.3)	0.0693
Neither Important nor Unimportant Barrier	29 (7.6)	33 (7.3)	
Important Barrier	288 (75.6)	366 (81.3)	
**Cost of palliative care consultation and treatment**			
Unimportant Barrier	132 (34.6)	93 (20.7)	<.0001
Neither Important nor Unimportant Barrier	77 (20.2)	84 (18.7)	
Important Barrier	172 (45.1)	273 (60.7)	
**Cultural differences between patients/families and physicians**			
Unimportant Barrier	94 (24.7)	66 (14.7)	0.0002
Neither Important nor Unimportant Barrier	67 (17.6)	67 (14.9)	
Important Barrier	220 (57.7)	317 (70.4)	
**Language differences between patients/families and physicians**			
Unimportant Barrier	130 (34.1)	132 (29.3)	0.0204
Neither Important nor Unimportant Barrier	86 (22.6)	80 (17.8)	
Important Barrier	165 (43.3)	238 (52.9)	

[a] Chi-Square Test

© 2023 McNeil MJ et al. *JAMA Network Open.*
