# Supplementary material for: Ideal vs Actual Timing of Palliative Care Integration for Children With Cancer in Latin America
Source: JAMA Netw Open. 2023 Jan 19;6(1):e2251496. doi: 10.1001/jamanetworkopen.2022.51496 (PMC9857245; doi:10.1001/jamanetworkopen.2022.51496)
Supplement: Supplement 2. — Nonauthor Collaborators [file jamanetwopen-e2251496-s002.pdf]

Supplemental Online Content: Nonauthor Collaborators

*Indicates required information. Only first name, last name, and suffix will appear in PubMed.

*Group Name(s): Assessing Doctors' Attitudes on Palliative Treatment (ADAPT) Latin America Study Group							
***First Name and Middle Initial(s)**	***Last Name**	***Suffix (eg, Jr, III)**	Academic Degrees	Institution	Location (city, state/province, country)	Role or Contribution, eg, chair, principal investigator	Group (if more than 1 Group listed in the byline) and/or Subgroup (eg, Steering Committee)
Florencia	Moreno						
Beatriz	Salas						
Rosa	Moreno						
Cinthia	Cespedes						
Gissela	Sanchez						
Soad	Fuentes						
Silvia	Rivas						
Pascale	Gaussant						
Clarissa	Aguilar						
Enrique	Lopez						
Roberta	Ortiz						
Diana	Cedeño						
Angelica	Samudio						
Jazmin	Servin						
Cecilia	Ugaz Olivares						
Fabiana	Morosini						
Alejandra	Chacon						
Lilliana	Barragan						
Ruth María	Castro						
Juliana	Lopera						
Miguel	Bayona						
Diana Lorena	Valencia						
Natalia	Perdomo						
Jeyni	Vega						
Wendy	Rivera						
Rosa Vanessa	Cabrera						
Emmanuel	Guerrero						
Soledad	Jimenez						
Libeth	Bosh						

Supplemental Online Content: Nonauthor Collaborators

*Indicates required information. Only first name, last name, and suffix will appear in PubMed.

***First Name and Middle Initial(s)**	***Last Name**	***Suffix (eg, Jr, III)**	Academic Degrees	Institution	Location (city, state/province, country)	Role or Contribution, eg, chair, principal investigator	Group (if more than 1 Group listed in the byline) and/or Subgroup (eg, Steering Committee)
Doris	Calle						
Sergio	Quintanilla						
Sandra	Felix						
Susana	Anaya						
Judith	Conejo Barrera						
Lilliana	Gallegos						
Martha	Valdez						
Valentine	Jimenez						
Susana	Juarez						
Berenice	Aguilar						
Adolfo	Pineda						
Isodoro	Romero						
Norma Arecil	Lopez Facundo						
Gelis	Ruis Piña						
Edgar Francisco	Ortiz						
Norma	Llamas						
Rosario	Batista						
