# Supplementary material for: Ideal vs Actual Timing of Palliative Care Integration for Children With Cancer in Latin America
Source: JAMA Netw Open. 2023 Jan 19;6(1):e2251496. doi: 10.1001/jamanetworkopen.2022.51496 (PMC9857245; doi:10.1001/jamanetworkopen.2022.51496)
Supplement: Supplement 3. — Data Sharing Statement [file jamanetwopen-e2251496-s003.pdf]

Data Sharing Statement

McNeil. Ideal vs Actual Timing of Palliative Care Integration for Children With Cancer in Latin America. *JAMA Netw Open*. Published January 19, 2023.
doi:10.1001/jamanetworkopen.2022.51496

Data
Data available: Yes
Data types: Deidentified participant data
How to access data: Data is available upon request.
When available: With publication

Supporting Documents
Document types: None

Additional Information
Who can access the data: Researchers whose proposed use of the data has been approved.
Types of analyses: Any specified purpose
Mechanisms of data availability: After approval of a proposal
